# Supplementary material for: Loss of immunity-related GTPase GM4951 leads to nonalcoholic fatty liver disease without obesity
Source: Nat Commun. 2022 Jul 16;13:4136. doi: 10.1038/s41467-022-31812-4 (PMC9288484; doi:10.1038/s41467-022-31812-4)
Supplement: Supplementary file 1 — Supplementary Information [file 41467_2022_31812_MOESM1_ESM.pdf]

Supplementary Information

Loss of immunity-related GTPase GM4951 leads to nonalcoholic fatty liver disease without obesity

Zhao Zhang[1,2,*], Yu Xun[1,2], Shunxing Rong[3,4], Lijuan Yan[1], Jeffrey A. SoRelle[1], Xiaohong Li[1], Miao Tang[1], Katie Keller[1], Sara Ludwig[1], Eva Marie Y. Moresco[1], Bruce Beutler[1,*]

[1]Center for the Genetics of Host Defense, University of Texas Southwestern Medical Center, Dallas, TX, 75390, USA.
[2]Division of Endocrinology, Department of Internal Medicine, University of Texas Southwestern Medical Center, Dallas, TX, 75390, USA.
[3]Center for Human Nutrition, University of Texas Southwestern Medical Center, Dallas, TX, 75390, USA.
[4]Department of Molecular Genetics, University of Texas Southwestern Medical Center, Dallas, TX, 75390, USA.

[*]Correspondence: Zhao.Zhang@UTSouthwestern.edu; Bruce.Beutler@UTSouthwestern.edu

This PDF file includes:
Supplementary Figures 1-8
Supplementary Table 1

a

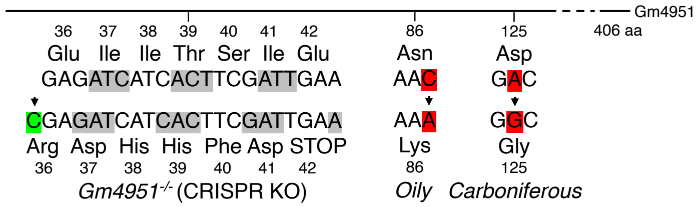

b

Birth ratio of *Gm4951*$^{+/-}$ breedings

Genotype	Pup numbers	Percentage
+/+	47	23.2%
Gm4951$^{+/-}$	105	51.7%
Gm4951$^{-/-}$	51	25.1%

Supplementary Fig. 1 *Gm4951* mutations and birth ratios of knockout mice. a Illustration of nucleotide and amino acid changes in *Oily*, *Carboniferous*, and *Gm4951* knockout mice. Green indicates an insertion; red indicates a substitution. **b** Number of pups with different genotypes generated from *Gm4951*$^{+/-}$ breedings.

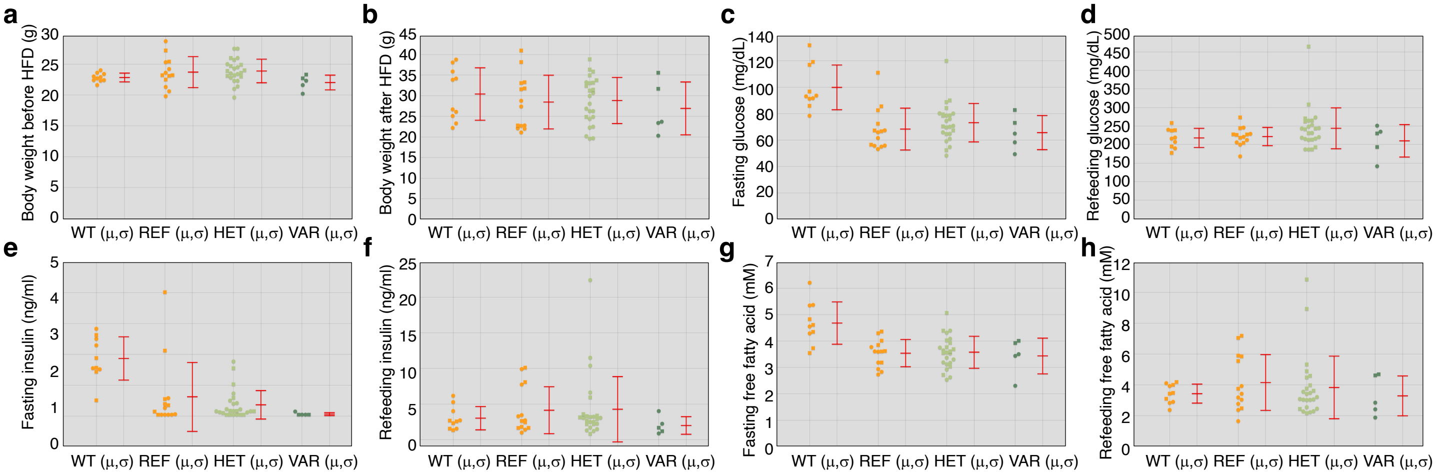

Supplementary Fig. 2 The *Oily* phenotype. a-h Body weight before HFD (**a**), body weight after 4 weeks on a HFD (**b**), overnight fasting glucose (**c**), glucose 2 h after refeeding (**d**), overnight fasting insulin (**e**), insulin 2 h after refeeding (**f**), overnight fasting free fatty acid (**g**), and free fatty acid 2 h after refeeding (**h**) in G3 mice were plotted vs. genotype at the *Oily* mutation site of *Gm4951* (n=10 mice in WT; n=14 mice in REF; n=25 mice in HET; n=5 mice in VAR). Data in **c**-**h** were measured after 2 weeks on a HFD. WT, C57BL/6J mice age-matched with G3 mice; REF, G3 mice homozygous for the reference allele of *Gm4951*; HET, G3 mice heterozygous for the reference allele and for the *Oily* allele; VAR, G3 mice homozygous for the *Oily* allele. Each data point represents one mouse. Mean (μ) and SD (σ) are indicated. Data are from one experiment.

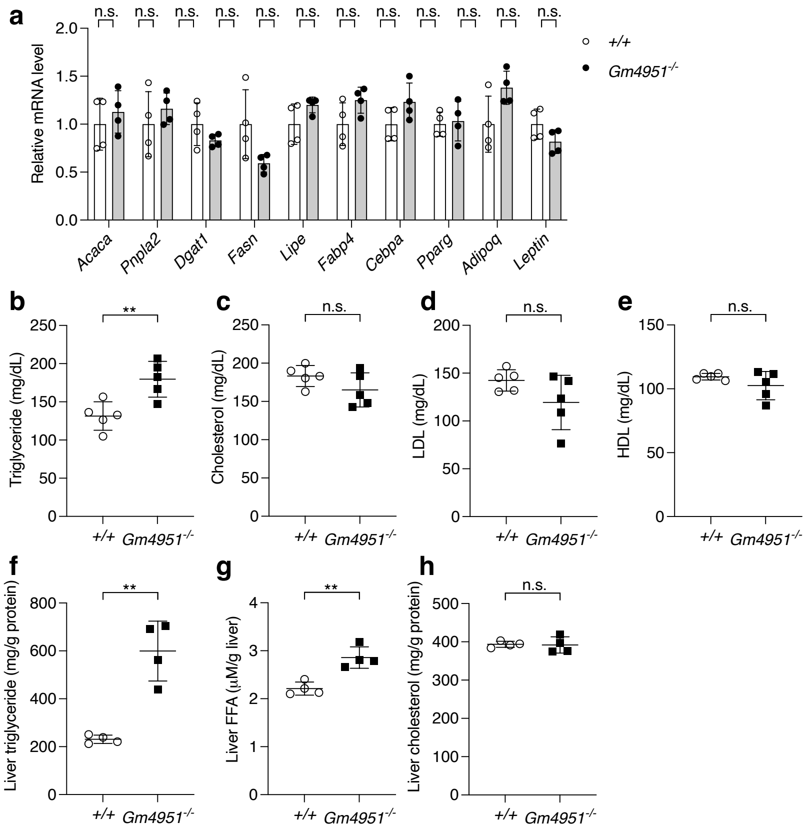

Supplementary Fig. 3 Lipid metabolism of adipose tissue, liver, and serum in WT and *Gm4951*$^{-/-}$ mice. a Relative mRNA level of different genes in the iWAT from WT and *Gm4951*$^{-/-}$ mice on HFD for 4 weeks (n=4 mice per genotype). Levels were normalized to *Polr2a* mRNA and then to levels in WT mice. **b**-**e** Fasting serum triglyceride (**b**), whole cholesterol (**c**), LDL cholesterol (**d**), and HDL cholesterol (**e**) of WT and *Gm4951*$^{-/-}$ mice on HFD for 4 weeks (n=5 mice per genotype). **f**-**h** Liver triglyceride (**f**), FFA (**g**), and cholesterol (**h**) of WT and *Gm4951*$^{-/-}$ mice on HFD for 4 weeks (n=4 mice per genotype). Each data point represents one mouse. Data are presented as means ± SD. P values were determined by two-tailed Student's t test. P values are denoted by ** $P < 0.01$; ns, not significant with $P > 0.05$. The exact P values of statistically significant groups are: 0.0069 (**b**), 0.0011 (**f**), 0.0026 (**g**). Data are representative of two independent experiments.

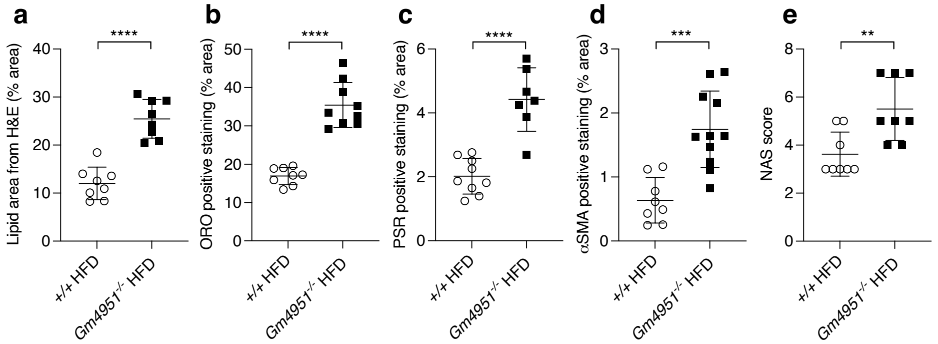

Supplementary Fig. 4 Quantification of histological stains in liver sections. a Quantification of lipid area in liver sections stained with H&E (n=8 images per genotype). **b-d**, Quantification of stained area in liver sections stained with ORO (n=8 images in *+/+*; n=9 images in *Gm4951*$^{-/-}$) (**b**), PSR (n=9 images in *+/+*; n=7 images in *Gm4951*$^{-/-}$) (**c**), and αSMA (n=8 images in *+/+*; n=11 images in *Gm4951*$^{-/-}$) (**d**). Data in **a-d** are expressed as a percentage of total area per image. **e** NAS score. Data points represent individual images. Data are presented as means ± SD. *P* values were determined by two-tailed Student's *t* test. *P* values are denoted by ** $P < 0.01$; *** $P < 0.001$; **** $P < 0.001$; ns, not significant with $P > 0.05$. The exact *P* values of statistically significant groups are: 4.29x10^{-6} (**a**), 5.094x10^{-7} (**b**), 2.505x10^{-5} (**c**), 0.0002 (**d**), 0.0051 (**e**). Data are from one experiment.

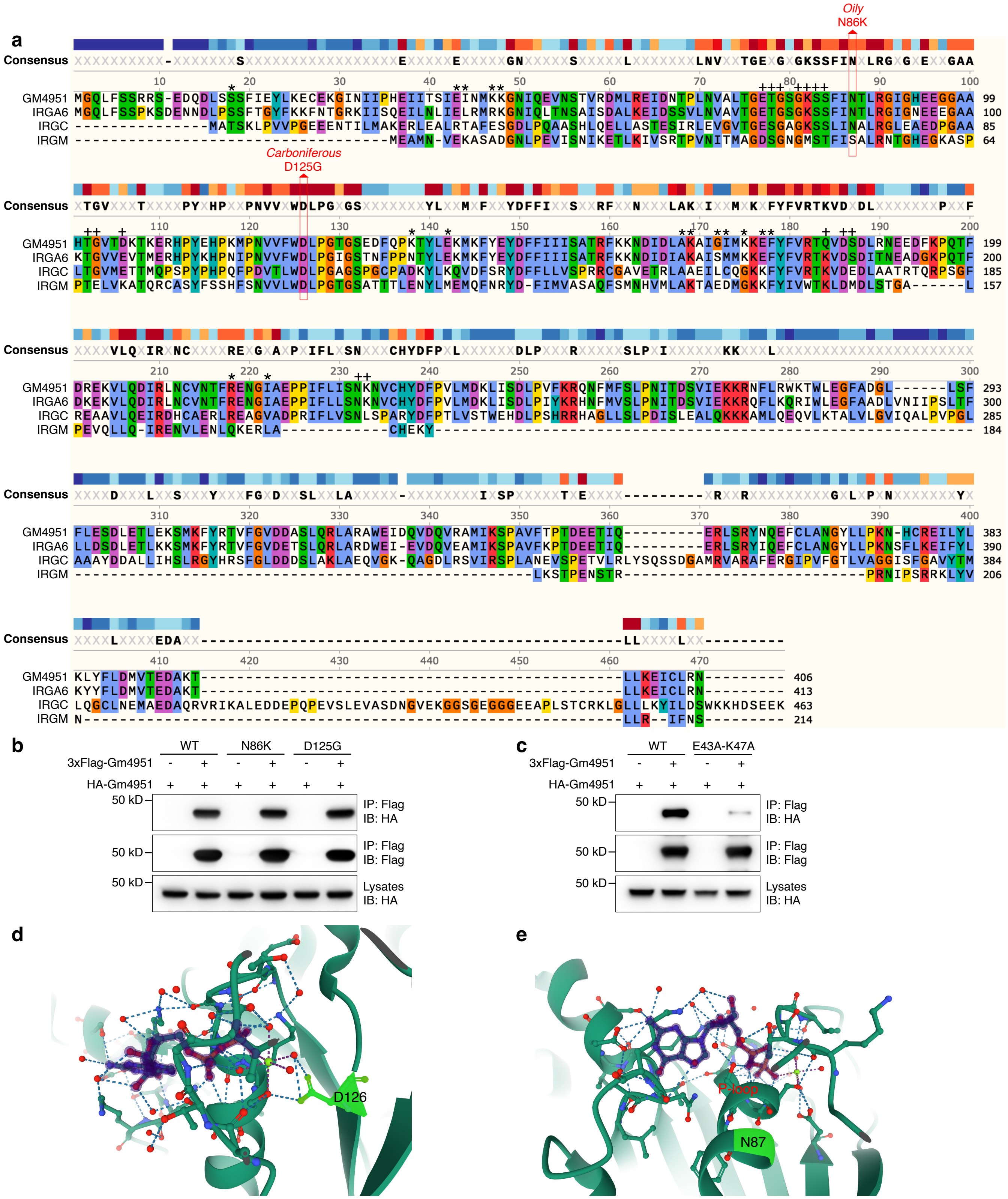

Supplementary Fig. 5 N86 and D125 are conserved between GM4951 and other IRG proteins. a Protein sequence alignment between mouse GM4951, mouse IRGA6, human IRGC, and human IRGM. According to IRGA6 structure[9], residues involved in dimer formation are marked with "*", and nucleotide interacting residues are marked with "+". **b-c** Immunoblots of immunoprecipitates (top and middle) or lysates (bottom) of 293T cells. **d-e** Crystal structure of IRGA6 (PDB# 1TPZ) and GDP visualized with Mol* to show the location of D126 (**d**), N87 and P-loop (**e**), Mg^{2+} (green ball), and GDP (highlighted in dark color). Data are representative of two independent experiments (**b-c**).

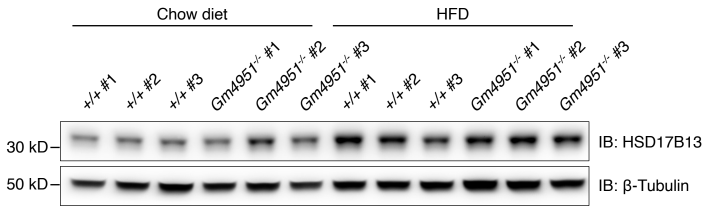

Supplementary Fig. 6 No change in total protein level of HSD17B13 in *Gm4951$^{-/-}$* liver. Immunoblots of liver lysates from *+/+* and *Gm4951$^{-/-}$* mice on chow diet or HFD for 4 weeks. Data are representative of two independent experiments.

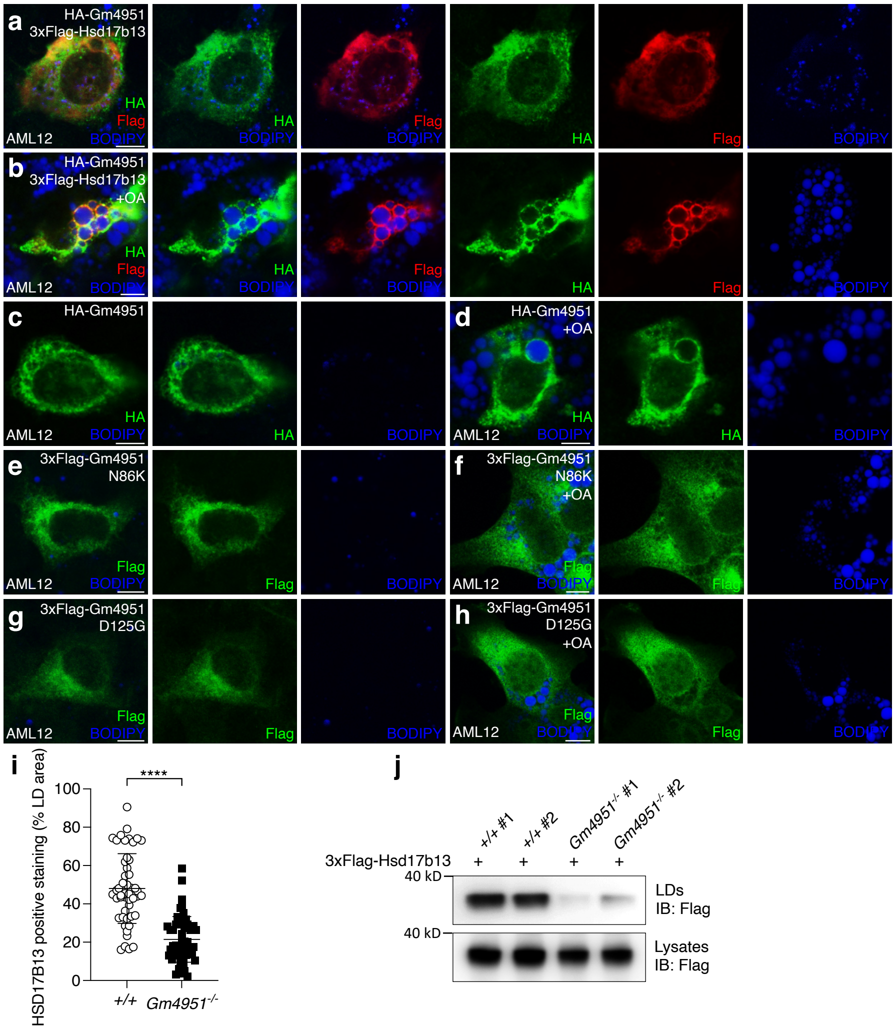

Supplementary Fig. 7 GM4951 specifically regulates the LD translocation of HSD17B13. a-b AML12 hepatocytes expressing HA-tagged Gm4951 and 3xFlag-tagged Hsd17b13 were immunostained with HA antibody (green), Flag antibody (red), and BODIPY (blue) to visualize LDs. 0.5 mM oleic acid for 24 h was used in +OA groups. **c-d** AML12 hepatocytes expressing HA-tagged Gm4951 without or with OA treatment were immunostained with HA antibody (green) and BODIPY (blue) to visualize LDs. **e-h** AML12 hepatocytes expressing 3xFlag-tagged Gm4951 mutants without (**e**, **g**) or with OA treatment (**f**, **h**) were immunostained with Flag antibody (green) and BODIPY (blue) to visualize LDs. **i** Quantification of the area with 3xFlag-HSD17B13 positive signal as a percentage of LD area in OA-treated primary hepatocytes of the indicated genotypes. LD area was defined by BODIPY positive signal. Each data point represents one LD (n=50 LDs in *+/+*; n=53 LDs in *Gm4951*$^{-/-}$). Data are presented as means ± SD. *P* value was determined by two-tailed Student's *t* test. *P* values are denoted by **** $P < 0.0001$. The exact *P* value is 3.927×10^{-14}. **j** Immunoblots of isolated LDs from 24h OA treated *+/+* and *Gm4951*$^{-/-}$ primary hepatocytes expressing 3xFlag-HSD17B13. Hepatocytes were isolated from mice on chow diet. Data are representative of two independent experiments.

a

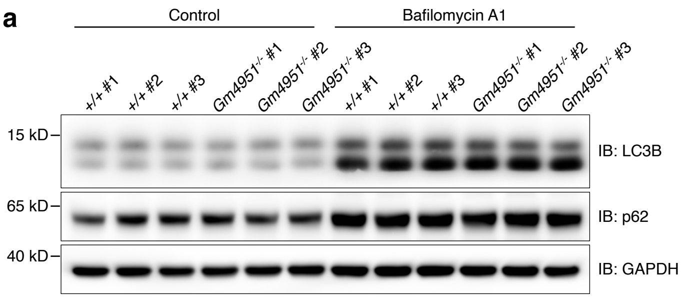

b

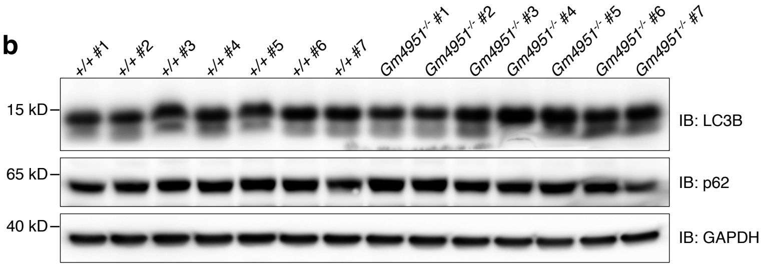

Supplementary Fig. 8 Autophagic flux in *Gm4951$^{-/-}$* primary hepatocytes and liver. a Immunoblots of lysates of primary hepatocytes isolated from *+/+* and *Gm4951$^{-/-}$* mice maintained on chow diet and treated *in vitro* with or without bafilomycin A1. **b** Immunoblots of liver lysates from *+/+* and *Gm4951$^{-/-}$* mice. Data are representative of two independent experiments.

Supplementary Table 1 Primers used for quantitative PCR.

Target name	Direction	Forward primer (5'-3')	Reverse primer (5'-3')
Gm4951	Forward	GTAGACTCAAGCCAAGAGCA	AGCAGAAACTTTAAACAGTGGG
Polr2a	Forward	CAAGATGCAAGAGGAGGAAGAG	TGTTGTCTGTCTGAGGTAAGTG
Slc27a1	Forward	CAGGCAAGGGCATGGATGAT	ATGCGGTAGTACCTGCTGTG
Cd36	Forward	ATTAATGGCACAGACGCAGC	TTCAGATCCGAACACAGCGT
Acaca	Forward	AGACTCACAACGAGATTTCAC	CATTTCATAAGACCACCGAC
Fasn	Forward	GATTCAGGGAGTGGATATTGTC	GAATGTTACACCTTGCTCCT
Elovl6	Forward	ACCGCAAGGCATTCATTTCC	AGTCGCTACGTGTTCTCTGC
Scd1	Forward	GTTCCGCCACTCGCCTAC	TGTAAGAACTGGAGATCTCTTGGA
Apob	Forward	TACTTCCACCCACAGTCCCCT	TGCTTTTTAGGGAGCCTAGCA
Mttp	Forward	AGCCCCACTCAGGCAATTC	GTTCACATCCGGCCACTAGG
Ppara	Forward	ATTTGCTGTGGAGATCGGC	GCTTTGGGAAGAGGAAGGTGT
Acot1	Forward	GGCTTGCACATGCGGTG	GAAAGGGTCCAGGTTCTGGG
Acox1	Forward	TCCAATCATGCGATAGTCCTGG	TCCCCAACAGTGATGCCTGG
Acadl	Forward	TGCACACATACAGACGGTGC	CATGGAAGCAGAACCGGAGT
3xFlag-Gm4951	Forward	TCTGCTATAACCTGCTGCGG	CCCATCTTGTCATCGTCATCCT
Pnpla2	Forward	TCTACTAAAGACCCTGCCTG	CAGACATTGGCCTGGATGAG
Dgat1	Forward	GAGGACGAGGTGCGAGAC	CAGACGATGGCACCTCAGAT
Lipe	Forward	CTATTCAGGGACAGAGGCAG	TAGTTCCAGGAAGGAGTTGAG
Fabp4	Forward	ACACCGAGATTTCCTTCAAACTG	CCATCTAGGGTTATGATGCTCTTC
Cebpa	Forward	CTCTGATTCTTGCCAAACTGAG	GACCCACTACTACATACACCC
Pparg	Forward	CTGAGGAGAAGTCACACTCTG	CGCTTCTTTCAAATCTTGTCTG
Adipoq	Forward	GCACTGGCAAGTTCTACTGCAA	GTAGGTGAAGAGAACGGCCTTGT
Leptin	Forward	TCATCCAAAGTAGAACCCTGTC	TGCTTTCCATCAAGTGTCCT
ACTB	Forward	AAGATCAAGATCATTGCTCCTC	ATACTCCTGCTTGCTGATCC
IRGC	Forward	TTCTGGACAGCTGGAAGAAACA	AGACTTAGTTTGTGGGTGAGGC
PPARA	Forward	GGATGTCACACAACGCGATTC	AGGCCTCGTAGATTCTCTTGG
ACOT1	Forward	GCTCAGTCATCCTGAGGTAA	GGAAAGAGGCCATGGAAAG
ACOX1	Forward	CTCCGTGCAGCCAGATTAGT	ATGTGCCTCACTTGCTCGAA
ACADL	Forward	CCCCTGTGTTTCGGACATGG	GAATGAGAACATCGCGCGGC
