# Supplementary material for: Loss of immunity-related GTPase GM4951 leads to nonalcoholic fatty liver disease without obesity
Source: Nat Commun. 2022 Jul 16;13:4136. doi: 10.1038/s41467-022-31812-4 (PMC9288484; doi:10.1038/s41467-022-31812-4)
Supplement: Supplementary file 2 — Reporting Summary [file 41467_2022_31812_MOESM2_ESM.pdf]

nature portfolio

Corresponding author(s): Zhao Zhang, Bruce Beutler

Last updated by author(s): May 12, 2022

Reporting Summary

Nature Portfolio wishes to improve the reproducibility of the work that we publish. This form provides structure for consistency and transparency in reporting. For further information on Nature Portfolio policies, see our Editorial Policies and the Editorial Policy Checklist.

Statistics

For all statistical analyses, confirm that the following items are present in the figure legend, table legend, main text, or Methods section.

n/a	Confirmed	
☐	☒	The exact sample size (*n*) for each experimental group/condition, given as a discrete number and unit of measurement
☐	☒	A statement on whether measurements were taken from distinct samples or whether the same sample was measured repeatedly
☐	☒	The statistical test(s) used AND whether they are one- or two-sided<br>*Only common tests should be described solely by name; describe more complex techniques in the Methods section.*
☒	☐	A description of all covariates tested
☐	☒	A description of any assumptions or corrections, such as tests of normality and adjustment for multiple comparisons
☐	☒	A full description of the statistical parameters including central tendency (e.g. means) or other basic estimates (e.g. regression coefficient) AND variation (e.g. standard deviation) or associated estimates of uncertainty (e.g. confidence intervals)
☐	☒	For null hypothesis testing, the test statistic (e.g. *F*, *t*, *r*) with confidence intervals, effect sizes, degrees of freedom and *P* value noted<br>*Give P values as exact values whenever suitable.*
☒	☐	For Bayesian analysis, information on the choice of priors and Markov chain Monte Carlo settings
☒	☐	For hierarchical and complex designs, identification of the appropriate level for tests and full reporting of outcomes
☒	☐	Estimates of effect sizes (e.g. Cohen's *d*, Pearson's *r*), indicating how they were calculated

Our web collection on statistics for biologists contains articles on many of the points above.

Software and code

Policy information about availability of computer code

Data collection	Immunostaining images were taken with a Zeiss LSM 880 inverted confocal using ZEN software.
Data analysis	GraphPad Prism 9 was used to generate graphs and perform statistical tests. ImageJ 1.53k was used to quantify stainings.

For manuscripts utilizing custom algorithms or software that are central to the research but not yet described in published literature, software must be made available to editors and reviewers. We strongly encourage code deposition in a community repository (e.g. GitHub). See the Nature Portfolio guidelines for submitting code & software for further information.

Data

Policy information about availability of data

All manuscripts must include a data availability statement. This statement should provide the following information, where applicable:
- Accession codes, unique identifiers, or web links for publicly available datasets
- A description of any restrictions on data availability
- For clinical datasets or third party data, please ensure that the statement adheres to our policy

The mass spectrometry proteomics data generated in this study have been deposited in the MassIVE repository with the accession code MSV000089462 (https://massive.ucsd.edu/ProteoSAFe/dataset.jsp?accession=MSV000089462). All other data are available in the main article or the Supplementary Information files. Source data are provided with this paper.

Field-specific reporting

Please select the one below that is the best fit for your research. If you are not sure, read the appropriate sections before making your selection.

☒ Life sciences ☐ Behavioural & social sciences ☐ Ecological, evolutionary & environmental sciences

For a reference copy of the document with all sections, see nature.com/documents/nr-reporting-summary-flat.pdf

Life sciences study design

All studies must disclose on these points even when the disclosure is negative.

Sample size	No pre-specified effect size was assumed, and in general three or more animals or replicates for each genotype or condition were used in experiments according to our previously published work and other published work in the field. This sample size was sufficient to demonstrate statistically significant differences in comparisons between two unpaired experimental groups by unpaired t-test.
Data exclusions	No data were excluded from any analyses.
Replication	The forward genetic screen was not repeated due to the limitation of mutagenesis by ENU, it is not possible to recreate the exact same mutation by a random mutagen ENU. Mass spectrometry was not repeated due to the nature of the study, all interesting hits were tested for interactions by CoIP to verify the mass spectrometry data. Lipid oxidation experiment was not repeated due to the limitation of radioactive experiments. Liver histology was performed one time due to liver samples were collected from multiple mice in each group. All other experiments were repeated at least twice and all attempts at replication were successful.
Randomization	The allocation of samples or animals to experimental groups was random.
Blinding	Blinding was not performed for this study because this is not a clinical trial and it is not required in the field.

Reporting for specific materials, systems and methods

We require information from authors about some types of materials, experimental systems and methods used in many studies. Here, indicate whether each material, system or method listed is relevant to your study. If you are not sure if a list item applies to your research, read the appropriate section before selecting a response.

Materials & experimental systems

n/a	Involved in the study	
☐	☒	Antibodies
☐	☒	Eukaryotic cell lines
☒	☐	Palaeontology and archaeology
☐	☒	Animals and other organisms
☒	☐	Human research participants
☒	☐	Clinical data
☒	☐	Dual use research of concern

Methods

n/a	Involved in the study	
☒	☐	ChIP-seq
☒	☐	Flow cytometry
☒	☐	MRI-based neuroimaging

Antibodies

Antibodies used	Immunostaining or immunohistochemistry:<br>The following primary antibodies were used in this study: mouse anti-Flag (M2, Sigma-Aldrich, 1:500), rabbit anti-HA [C29F4, Cell Signaling Technology (CST), 1:800), anti-AIF (D39D2, CST, 1:400), anti-PDI (C81H6, CST, 1:100), anti-EEA1 (C45B10, CST, 1:200), anti-RCAS1 (D2B6N, CST, 1:200), anti-SMA (D4K9N, CST, 1:400), and anti-PLIN2 (EPR3713, Abcam, 1:200). The following secondary antibodies were used in the study: Alexa Fluor 488 Goat anti-Mouse IgG (H+L) (115-545-166, Jackson ImmunoResearch, 1:500), Alexa Fluor 488 Goat anti-Rabbit IgG (H+L) (115-545-144, Jackson ImmunoResearch, 1:500), Rhodamine Red-X Goat anti-Mouse IgG (H+L) (115-295-146, Jackson ImmunoResearch, 1:500), and Rhodamine Red-X Goat anti-Rabbit IgG (H+L) (111-295-144, Jackson ImmunoResearch, 1:500).<br><br>Western blot:<br>The following primary antibodies were used in this study: mouse anti-HA (HA-7, Sigma-Aldrich, 1:5000), anti-Flag (M2, Sigma-Aldrich, 1:5000), rabbit anti-Gapdh (D16H11, CST, 1:2000), anti-β-tubulin (9F3, CST, 1:2000), anti-p62 (D6M5X, CST, 1:1000), anti-LC3B (D3U4C, CST, 1:1000), anti-HSD17B13 (gift from Dr. Helen Hobbs, 1:2000). The following secondary antibodies were used in this study: Goat anti-Mouse IgG (H+L) HRP (115-035-146, Jackson ImmunoResearch, 1:5000), Goat anti-Mouse IgG (light chain specific) HRP (115-035-174, Jackson ImmunoResearch, 1:5000), and Goat anti-Rabbit IgG (H+L) HRP (111-035-144, Jackson ImmunoResearch, 1:5000).
Validation	All commercially available antibodies used in this study were previously validated for the specific application by the companies from which we purchased them. Statements for validation of antibodies can be found from these manufacturers' websites:

Sigma-Aldrich (https://www.sigmaaldrich.com/US/en)
Cell Signaling Technology (https://www.cellsignal.com)
Abcam (https://www.abcam.com)
Jackson ImmunoResearch (https://www.jacksonimmuno.com)

HSD17B13 antibody was validated for western blot to detect mouse HSD17B13 by Dr. Helen Hobbs's lab at UT Southwestern Medical Center.

Eukaryotic cell lines

Policy information about cell lines

Cell line source(s)	HEK293T, AML12, SW1088, SW872, SK-LMS-1, HepG2, THP-1 cells and primary pre-adipocytes were purchased from ATCC. Human primary hepatocytes were purchased from Zen-Bio.
Authentication	Cell lines were not authenticated.
Mycoplasma contamination	The cell lines were directly used in experiments and were not tested for mycoplasma contamination.
Commonly misidentified lines (See ICLAC register)	This study did not involve commonly misidentified lines.

Animals and other organisms

Policy information about studies involving animals; ARRIVE guidelines recommended for reporting animal research

Laboratory animals	All mice were housed at room temperature (18-23°C) with 40-60% humidity in a 12h light/12h dark cycle. Six- to thirty-week old male and female mice on a C57BL/6J background were used in experiments.
Wild animals	This study did not involve wild animals.
Field-collected samples	This study did not involve field-collected samples.
Ethics oversight	All experiments in this study were approved by the University of Texas Southwestern Medical Center Institutional Animal Care and Use Committee.

Note that full information on the approval of the study protocol must also be provided in the manuscript.
