# Supplementary material for: Loss of immunity-related GTPase GM4951 leads to nonalcoholic fatty liver disease without obesity
Source: Nat Commun. 2022 Jul 16;13:4136. doi: 10.1038/s41467-022-31812-4 (PMC9288484; doi:10.1038/s41467-022-31812-4)
Supplement: Supplementary file 3 — Description of Additional Supplementary Files [file 41467_2022_31812_MOESM3_ESM.pdf]

Title: Supplementary Data 1.
Description: Proteins identified by mass spectrometric analysis of immunoprecipitates from WT and 3xFlag-Gm4951 mouse livers.
